# Chemical composition and biological activity of extracts from fruiting bodies and mycelial cultures of *Fomitopsis betulina*

**DOI:** 10.1007/s11033-018-4420-4

**Published:** 2018-10-13

**Authors:** Katarzyna Sułkowska-Ziaja, Agnieszka Szewczyk, Agnieszka Galanty, Joanna Gdula-Argasińska, Bożena Muszyńska

**Affiliations:** 1 Department of Pharmaceutical Botany, Faculty of Pharmacy, Jagiellonian University Medical College, Medyczna 9, 30-688 Kraków, Poland; 2 Department of Pharmacognosy, Faculty of Pharmacy, Jagiellonian University Medical College, Medyczna 9, 30-688 Kraków, Poland; 3 Department of Radioligands, Faculty of Pharmacy, Jagiellonian University Medical College, Medyczna 9, 30-688 Kraków, Poland

**Keywords:** *Fomitopsis betulina*, Mycelial culture, Cytotoxic activity, Anti-inflammatory activity, Betulin, Betulinic acid

## Abstract

*Fomitopsis betulina* (Bull.) B.K. Cui, M.L. Han & Y.C. Dai has been used for medicinal purposes for over 5000 years. Numerous studies have confirmed the biological activity of compounds found in this species. The purpose of this study was a comparative analysis of selected groups of metabolites in the extracts from fruiting bodies and mycelial cultures. Phenolic acids (syringic, gallic, *p*-hydroxybenzoic, 3,4-dihydrophenylacetic), indole compounds (l-tryptophan, 5-hydroxy-l-tryptophan, 5-methyltryptamine), sterols (ergosterol, ergosterol peroxide, hexestrol, cholecalciferol), and triterpenes (betulinic acid, betulin) were determined quantitatively by high performance liquid chromatography with UV–Vis/DAD detection, while fatty acids were assessed with the gas chromatography method. Cytotoxic activity against selected human cancer cell lines was determined using the lactate dehydrogenase test. Anti-inflammatory activity was evaluated on lipopolysaccharide activated A549 cells. Those extracts with anti-inflammatory activity were evaluated for their inhibition of pro-inflammatory enzymes. The mycelium extract exhibited significant cytotoxic activity against prostate cancer cells, while the fruiting body extract indicated a moderate effect on the viability of melanoma and prostate cancer. Incubation of lung epithelial cells with biomass extract significantly decreased cyclooxygenase-2 levels compared to LPS activated A549 cells. This paper is the first report of a comparative quantitative analysis of the metabolites in mycelial cultures and fruiting bodies. In addition, a novel element of this study is its comparison of the cytotoxic and anti-inflammatory activity of the obtained extracts. The results of comparing the composition and activity of mycelium and fruiting bodies shows that the cultures could be proposed as a potential biotechnological source for selected biologically active compounds.

## Introduction

*Fomitopsis betulina* (Bull.) BK Cui, ML Han and YC Dai [formerly *Piptoporus betulinus* Bull. P. Karst.—(birch polypore)] from the family of *Fomitopsidaceae* (Basidiomycota) is a common species of arboreal mushroom [1]. A characteristic feature of this species is the colonization of both living and dead individuals of *Betula* sp. genus. This is an inedible species; however, young fruiting bodies are suitable for consumption [2]. The oldest evidence of the use of *F. betulina* fruiting bodies by humans is the discovery of fragments with the remains of the ice man—Ötzi, who lived 5300 years ago (3300 BC). This fact allows the conclusion that the fruiting bodies of this species were used for healing purposes [3]. The fruiting bodies of *F. betulina* have also been widely used in folk medicine due to their antimicrobial properties that accelerate wound healing. The fruiting bodies have also been employed as a laxative and adjuvant in stomach disorders [3]. Most of the potential therapeutic applications described above have been confirmed in scientific studies. Aqueous extracts and pentacyclic triterpenes isolated from fruiting bodies exhibit immunomodulatory and anticancer effects [4]. Ethanol-acetate extract and single triterpene derivatives of lanostane exhibit anti-inflammatory activity [5]. Another documented effect is its antioxidant activity and the ability to inhibit hyaluronidase, an enzyme depolymerizing hyaluronic acid and causing the inhibition of acetylcholinesterase, which is used in the symptomatic treatment of Alzheimer’s disease [6].

An alternative method to obtain secondary metabolites with therapeutic properties from fruiting bodies is to use the biosynthetic capability of mycelial cultures. The main advantage of mycelial cultures is their independence from environmental conditions and their ability to continuously produce high-quality material; however, there may be differences in the chemical composition of mycelium and fruiting bodies [7].

The chemical composition of biomass extracts from mycelial cultures of *F. betulina* has so far only been studied to a limited extent. However, the anticancer properties of the extracts have been proven [8].

A challenge facing modern science are the so-called civilization diseases, which include cancer diseases. Inflammation, a factor often preceding the formation of a tumor, plays an important role in the process of oncogenesis and cancer progression. Studies have proved the existence of a feedback mechanism that allows chronic inflammation to be connected with metastasis. Inflammatory responses play decisive roles at different stages of tumor development, including initiation, promotion, malignant conversion, invasion, and metastasis [9]. This observation led us to study the cytotoxic and anti-inflammatory potential of extracts from fruiting bodies and from mycelial cultures of *F. betulina*.

The present study is the first to present a comparative analysis of groups of bioactive metabolites in extracts from fruiting bodies and from mycelial cultures. The multidirectional biological activity of extracts from fruiting bodies and biomass of *F. betulina* has so far been explained mainly by the activity of polysaccharides and triterpenes. A comprehensive understanding of the qualitative and quantitative profiles of the groups of metabolites (sterols, indole derivatives, phenolic acids and fatty acids) was established using chromatographic methods. In addition, for the first time, the cytotoxic and anti-inflammatory activities of the obtained extracts were compared using in vitro methods.

## Materials and methods

The fruiting bodies were collected in 2014 in the mixed forests of northern Poland. Representative samples were deposited at the Department of Pharmaceutical Botany, Jagiellonian University Medical College, Kraków.

Mycelial cultures were isolated from the internal part of the fruiting bodies. Pieces of sporocarps were surface-sterilized with 70% ethanol for 2 min. Then, these were rinsed three times with sterile water and placed on Oddoux medium [10]. Initial cultures were grown for three weeks at 22 ± 2 °C under a 16/8 h light/dark period. The experimental cultures were maintained as submerged cultures on Oddoux medium. Medium was inoculated with 3-week-old initial cultures. The cultures (three series) were maintained for 3 weeks, under the same light and temperature conditions as the initial cultures, on an Alltel rotary shaker operating at 140 rpm with a vibration amplitude of 35 mm. After separation from the medium, the obtained mycelium was frozen and lyophilized (lyophilizer Freezone 4.5, Labconco, USA) at − 40 °C. After lyophilization, materials were ground in a mortar and used for chemical analyses.

### Extraction and HPLC analysis of phenolic acids

Two grams of material was extracted by boiling methanol (at 67.4 °C) for 2 h under a reflux condensor. Combined extracts (200 mL) were concentrated to dryness using a rotary vacuum evaporator (Büchi) at 40 °C. Residues were dissolved in 10 mL of methanol. The filtered sample (Millipore PTFE membrane, 0.45 µm) was injected (20 µL) in an HPLC column. The HPLC method was followed according to a procedure described previously [11]. HPLC analyses were conducted using a Hitachi HPLC VWR apparatus: pump L-2130, RP-18e column (250 mm × 4 mm, 5 µm) thermostated at 25 °C, and diode array detector (DAD) L-2455 at UV range 200–400 nm.

The gradient program was as follows: 0–20 min, 0% B; 20–35 min, 0–20% B; 35–45 min, 20–30% B; 45–55 min, 30–40% B; 55–60 min, 40–50% B, 60–65 min, 50–75% B; and 65–70 min, 75–100% B, with a hold time of 15 min, at 25 °C. The flow rate was 1.0 mL/min. Comparison of UV spectra at λ = 254 and retention times with standard compounds enabled identification of phenolic acids present in the analyzed samples. Phenolic acid standards were purchased from Fluka (Chemie AG) and Sigma (St. Louis, USA).

### Extraction and GC analysis of fatty acids

One gram of material was extracted with a chloroform–methanol solution, 2:1 (v/v). Fatty acid methyl esters (FAME) were synthesized using 20% BF_3_ in methanol at 100 °C. FAME analyses were performed using gas chromatography (GC) on an Agilent 6890N with a J and W DB-23 capillary column (60 m, ID 0.25 mm, 0.25 µm) and FID detector. Chromatography parameters—FID 260 °C, injector 250 °C, split ratio 50:1, oven 140 °C for 5 min—ranged from 140 to 190 °C at 4 °C/min, 190 °C for 15 min, and from 190 to 240 °C at 2.75 °C /min, 240 °C for 4 min, carrier gas—helium. For the identification of fatty acids, retention times of FAME standards from Supelco (47801) were used. Peak areas were measured with an integrator (ChemStation). The results for fatty acid (FA) composition, total saturated fatty acids (SFA), monounsaturated fatty acids (MUFA) and polyunsaturated fatty acids (PUFA) of the samples were expressed as relative % of total fatty acids.

### Extraction and HPLC analysis of sterols

Five grams of material was extracted with a mixture of methanol/dichloromethane, 75:25 (v/v). The mixture was sonicated at 40 kHz for 10 min. After 2 h, the extract was centrifuged at 12,000 rpm for 5 min and decanted. The extraction procedure was repeated twice and obtained extracts were mixed and evaporated under reduced pressure.

The filtered sample (Millipore PTFE membrane 0.45 µm) was injected (20 µL) into the HPLC column. Analyses were conducted using a Hitachi HPLC VWR liquid chromatograph (Merck, Germany), as described above.

The HPLC method was followed according to the procedure developed by Yuan [12] with our own modifications concerning the gradient procedure. The mobile phase consisted of solvent A: methanol/water 80:20 (v/v), and solvent B: methanol/dichloromethane 75:25 (v/v The gradient program was as follows: 0–10 min, 80:20% B; 10–35 min, 40–60% B; 35–50 min, 0–100% B; 50–55 min, 80–20% B; with a hold time of 15 min, at 25 °C). The flow rate was 1.0 mL/min. The chromatographic peaks were recorded at a wavelength of 280 nm. Sterol standards were purchased from Fluka (Chemie AG).

### Extraction and HPLC analysis of triterpenoids

One gram of material sample was mixed with 10 mL methanol 95% containing 1% HCl conc. and then homogenized under sonication for 15 min. After 30 min, the extract was filtered through a paper and Millipore PTFE membrane (0.45 µm).

The HPLC method was followed according to the procedure developed by Holonec [13]. The filtered sample was injected (20 µL) into the HPLC column. HPLC analyses were conducted using a Hitachi HPLC VWR liquid chromatograph (Merck, Germany), as described above. An isocratic separation was applied, for which the mobile phase consisted of acetonitrile: water, 9:1, flow 1 mL/min. at temperature 25 °C and wavelength at 210 nm. Triterpenoid standards were purchased from Sigma (St. Louis, USA).

### Determination of indole derivatives

Five grams of material was extracted with 100 mL of methanol for 2 h in a magnetic stirrer. The obtained extracts were combined and concentrated to dryness. The HPLC method was carried out according to the procedure described by Muszyńska [14]. Briefly, the conditions were as follows: Hitachi HPLC; pump L-7100; column Purospher RP-18 (250 mm × 4 mm, 5 µm). Isocratic separation was used, and the mobile phase was: methanol: water: ammonium acetate 15:14:1 (v/v/v); flow 1 mL/min. Chromatographic peaks were recorded at a wavelength of 280 nm. Indole standards were purchased from Sigma (St. Louis, USA).

### Cytotoxicity determination

Five grams of material was extracted three times with 100 mL of methanol for 24 h in a magnetic stirrer. The obtained extracts were combined and concentrated to dryness.

A cytotoxicity determination was performed on human cancer cells and their corresponding normal cells. Prostate cancer and melanoma lines, grouped into panels, were used for this purpose. The prostate panel comprised DU145 cancer cells as well as normal epithelial cells of the prostate—PNT-2. The melanoma panel comprised the WM795 and A375 cell lines differing in metastatic potential as well as the reference normal cells of skin fibroblasts—BJ. All the cell lines were commercial (ATCC) and remained in a continuous culture at the Department of Pharmacognosy, Jagiellonian University Medical College (Poland). Cells were grown on media (DMEM F12, DMEM high glucose) with 10% bovine serum and a mixture of antibiotics in an incubator with 5% CO_2_ at 37 °C and constant humidity. The cytotoxic activity of the tested extracts was measured with the LDH viability test, according to a procedure described previously [15]. Each experiment was repeated three times. The cytotoxicity of the samples was measured as follows: % cytotoxicity = [(Asample − Aspont): (Amax − Aspont)] × 100 where Asample is the absorbance value for the cells treated with the tested substances, Aspont is the value for the spontaneous LDH release and Amax the value in lysed cells in the presence of Triton × 100.

### Anti-inflammatory activities

#### Cell cultures

A549 Human Lung Carcinoma Epithelial Cells (CCL-185, ATTC, Manassas, VA, USA) were cultured as described previously [16]. Cells were activated by LPS 1 µg/mL for 24 h. After this time, lung epithelial cells were incubated with 10 µL or 25 µL of methanolic extract from biomass and fruiting bodies for 24 h.

#### Western blot

Cell lysates were prepared and subjected to 10% SDS–polyacrylamide gel electrophoresis, as described earlier [16]. The experiments used primary antibodies anti-cyclooxygenase 2 (COX-2) and anti-β-actin (GeneTex Inc., Irvine, CA, USA), diluted 1:1000 and the secondary antibody anti-rabbit IgG (HRP) diluted 1:2000 (ThermoFisher Scientific, Waltham, MA, USA). The integrated optical density of the bands was quantified using a Chemi Doc Camera with Image Lab software (Bio-Rad, Hercules, CA, USA).

### Statistical analysis

Values are presented as means ± SD. All experiments were performed four times. Using one way ANOVA with Tukey–Kramer post hoc method of multiple comparisons, statistical analysis of the data was performed. The value p < 0.05 was accepted as the level of statistical significance.

## Results and discussion

The use of mycelial cultures of macrofungi for experimental and medical purposes is a promising and reproducible method that enables efficient production of fungal biomass and its metabolites [17]. Differences in the content of bioactive compounds in fruiting bodies derived from natural sites may result not only from genetic differences between populations, but also from different habitats and environmental conditions during growth and development. Differences in the amounts of bioactive compounds such as polysaccharides, terpenoids, steroids and phenolic compounds are much lower under the controlled, reproducible conditions of mycelial cultures.

After a 3-week growth cycle, the biomass increments were on average 9.5 g dry mass per liter. Obtained biomass increments and growth dynamics did not differ from the results obtained previously by other researchers [18]. The applied methods for extraction and HPLC analysis provided the opportunity to obtain optimal conditions for the qualitative and quantitative determination of selected compounds in the examined material. Previous analysis of the chemical composition of fruiting bodies of *F. betulina* had demonstrated that they mainly contain carbohydrates, terpene compounds, sterols, fatty acids and aromatic compounds [19]. Chemical biomass composition has so far been studied only to a limited extent.

In contrast, biological activity studies have focused on extracts. Cyranka et al. demonstrated that the type of mycelial extract has an effect on the growth and viability of colon adenocarcinoma cells [8]. Moreover Pleszczyńska et al. showed the anti-proliferative and anti-immigration properties of water and ethanol extracts from fruiting bodies against human cancer cells [20].

The total content of phenolic acids in the biomass obtained from mycelial cultures was 22.56 mg/100 dry mass (DM.). The total content of phenolic acids in fruiting bodies was 37.08 mg/100 DM. Syringic, gallic, 5-hydroxybenzoic and 3,4-dihydrophenylacetic acids were determined in mycelium and fruiting bodies (Table 1).

**Table 1:** Quantitative comparison of studied groups of compounds in fruiting bodies and biomass of *Fomitopsis betulina*

Name of compounds	Amounts of compounds [mg/100 g DM]	
	Fruit bodies	Biomass from mycelial cultures
Non hallucinogenic indole derivatives		
l-Tryptophan	2.03 ± 0.83^a^	1.34 ± 0.28^a^
5-Hydroxy-l-tryptophan	3.05 ± 1.02^a,b^	2.74 ± 0.62
5-Methyltryptamine	5.25 ± 1.6 ^a,b^	3.99 ± 0.95^a^
Phenolic acids		
Syringic acid	0.99 ± 0.86^a^	1.08 ± 0.74^a^
Gallic acid	7.55 ± 1.30^a,b^	4.02 ± 1.01^a,b^
5-Hydroxybenzoic acid	10.88 ± 1.20^a,b,c^	9.11 ± 1.29^a,b^
3,4-Dihydrophenylacetic acid	6.55 ± 0.82^a,c^	8.04 ± 0.98^a,b^
Triterpenes		
Betulin	0.00209 ± 0.00102	0.0006 ± 0.00022
Betulinic acid	0.00085 ± 0.00068	0.00031 ± 0.00037
Sterols		
Ergosterol	103.99 ± 2.63^a^	41.29 ± 2.87^a^
Hexestrol	3.55 ± 0.71^a,b^	4.01 ± 1.75^a,b^
Ergosterol peroxide	11.88 ± 0.87^a,b,c^	17.11 ± 1.50^a,b,c^
Cholecalciferol	6.55 ± 0.04^a,b,c^	0.31 ± 0.30^a,c^

Data are presented as the mean ± SD (standard deviation); n = 3 repetitions. Tukey test was used to reveal the differences between paired groups of elements in rows, the same letters (a, b, c) are marked for which the content differences are statistically significant (for *p* values < 0.05); (GraphPad InStat)

Phenolic acids identified both in fruiting bodies and mycelial culture show a broad spectrum of biological activity of antioxidant, antibacterial, antiviral, antifungal and anti-inflammatory nature. Particularly noteworthy is the antioxidant activity of phenolic acids. A deficiency of these acids induces the human body into a state of chronic oxidative stress, the consequence of which may be chronic inflammation that leads to the development of many diseases.

Syringic acid determined in mycelial cultures also exhibits a choleretic activity, while in turn gallic acid possesses antiseptic, astringent and antiperspirant activity.

The most common phenolic acids in fruiting bodies of higher mushrooms include protocatechuic acid, gallic acid, *p*-hydroxybenzoic acid, gentisic acid, caffeic acid, and syringic and vanillic acids. Veratryl, *p*-coumaric, caffeic, ferulic or cinnamic acids are less common [21]. Phenolic acids are compounds important for the survival of fungal organisms, as evidenced by the various biogenetic pathways leading to their formation. Similar to the situation in plants, phenolic acids play a defensive role against parasites and microorganisms in mushrooms. Their insecticidal, anti-bacterial and anti-microbial properties are known [22].

In both mycelium and fruiting bodies eleven fatty acids were identified. The results of the analysis are presented in Table 2.

**Table 2:** Quantitative comparison of fatty acids in fruiting bodies and biomass of *Fomitopsis betulina*

Common names of fatty acids	Lipid numbers	Amounts of fatty acids [%]	
		Extract from fruit bodies	Extract from in vitro cultures
SFA			
Capric acid	C10:0	5.42 ± 1.76^a^	1.06 ± 0.01^a^
Lauric acid	C12:0	14.13 ± 1.75^a,b^	0.59 ± 0.09^a,b^
Myristic acid	C14:0	1.29 ± 0.1^b,c^	0.67 ± 0.29 ^a,c^
Pentadecanoic acid	C15:0	29.53 ± 2.73^a,b,c,d^	0.91 ± 0.47^a,d^
Palmitic acid	C16:0	13.82 ± 2.50^a,c,d,e^	35.8 ± 1.53^b,c,d,e^
Stearic acid	C18:0	41.60 ± 3.03^a,b,c,d,e^	17.01 ± 0.98^b,c,d,e^
MUFA			
Myristoleic acid	C14:1n5	1.71 ± 0.87^a^	15.30 ± 2.96^a^
Palmitooleic acid	C16:1c	2.82 ± 1.84^b^	0.42 ± 0.64^a^
Oleic acid	C18:1w9	40.78 ± 2.46^a,b,c^	2.86 ± 0.08^a^
Cis-10-pentadecanoic acid	C15:1	0.43 ± 0.57^c^	0.55 ± 0.53^a^
PUFA			
Linoleic acid	C18:2w6	1.1 ± 0.95	25.3 ± 0.82

Data are presented as the mean ± SD (standard deviation); n = 3 repetitions. Tukey test was used to reveal the differences between paired groups of elements in rows, the same letters (a,b,c,d,e) are marked for which the content differences are statistically significant (for *p* values < 0.05); (GraphPad InStat)

The amounts of individual compounds were within a wide range—from about 35.8% (palmitic acid) to 0.4% (palmitooleic acid)—in the extracts obtained from biomass. In turn, in extracts from fruiting bodies, the amounts range from 41.6% (stearic acid) to 0.4% (cis-10-pentadecanoic acid). The amount of palmitic acid is 2.5-fold higher, and that of myristoleic acid is ninefold higher in extracts obtained from biomass than in extracts from fruiting bodies. However, most interesting is the fact that the concentration of linoleic acid is up to 23-fold higher in extracts obtained from mycelial biomass cultures than in extracts of fruiting bodies.

Linoleic acid (LA) belongs to the group of essential fatty acids (EFAs). It is a precursor of arachidonic acid, the component of bile and octen-3-ol which gives mushrooms their characteristic aroma. In addition, LA reduces the total fat in the blood and, as a result, reduces the risk of cardiovascular diseases [15, 16]. A high intake of dietary n-6 polyunsaturated fatty acid contributes to excess chronic inflammation. For linoleic acid (LA), as a precursor of long-chain fatty acids from the n-6 family, there is no evidence that a high intake of LA with the diet influences the level of inflammatory markers in blood [23].

Fatty acids contained in mushrooms may support anti-inflammatory processes in the human organism, due to the high content of unsaturated fatty acids [24, 25].

Polyunsaturated fatty acids (PUFAs) are precursors of eicosanoids, signaling molecules necessary for proper regulation of cellular processes in muscles, blood vessels, nerve cells and in the immune system. Eicosanoids provide a balance between inflammatory and anti-inflammatory processes [26]. PUFAs include the n-3, n-6 and n-9 series acids. Maintaining the correct proportions of fatty acids from the n-3 to n-6 series in the diet is crucial in preventing the development of cardiovascular diseases or cancers. α-Linolenic acid (ALA) is an essential ingredient in normal nutrition; it is a precursor to the long-chain PUFAs of the n-3 series. It also exhibits anti-inflammatory activity [27].

Sterols determined in mycelial cultures of *F. betulina* included ergosterol (41.29 mg/100 g D.), cholecalciferol (0.30 g DM), ergosterol peroxide and hexestrol. The results of the quantitative analysis are presented in Table 1.

Sterols are common ingredients of fruiting bodies of most Basidiomycota representatives. The most common is ergosterol. Ergosterol and its peroxide are essential for the proper development of the hyphae of higher fungi. Ergosterol (provitamin D_2_) is one of the main components of fungal cell membranes. It has anti-cancer and immunostimulating properties and is also a precursor of cortisol, an adrenal hormone with anti-inflammatory activity.

Numerous studies have shown that ergosterol and its peroxidation products (ergosterol peroxide) show a therapeutic effect by reducing the pain related to inflammation, reducing the incidence of cardiovascular disease and inhibiting the action of cyclooxygenase enzymes (COX) [28]. Ergosterol present in the fruiting bodies of edible mushrooms (e.g., *Imleria badia* and *A. bisporus*) has an anti-inflammatory and anticancer activity [19]. Anti-inflammatory properties in the mouse model have also been demonstrated for *Lentinula edodes* extract, which is rich in ergosterol. Supplementation with *L. edodes* extract enriched with vitamin D using UV-B radiation in C57B1/6 mice with mitogen-induced (concanavalin A) liver inflammation causes a significant reduction in liver damage. The histopathological image of tissues is improved and also the plasma level of transaminases and INF-γ decreased. In addition, the anti-inflammatory effect of vitamin D and fungal extract is synergistic [29].

In the study material, three indole derivatives were determined: 5-hydroxy-l-tryptophan, 5-methyltryptamine and l-tryptophan in the both type of extracts.. Quantitative results are presented in Table 1. The largest amount was found in the case of 5-methyltryptamine (3.99 mg/100 g DM in mycelial cultures), while the smallest was for l-tryptophan (1.34 mg/100 g DM on in mycelial cultures). The total content of indole compounds in the biomass was 8.07 mg/100 g DM and in the fruiting bodies 10.33 mg/100 g DM.

l-tryptophan and 5-hydroxy-l-tryptophan identified in mycelial cultures are observed in in vitro cultures of numerous edible species of *Sarcodon imbricatus, I. badia* and *Cantharellus cibarius* [30–32]. The concentrations of these two indole derivatives are higher in the mycelial cultures of the above-mentioned species than in the cultures of *F. betulina*, with the exception of *I. badia* cultures. The concentration of 5-hydroxytryptophan in the mycelium of this species was lower than 1 mg/100 g DM, whereas in *F. betulina* its content was 2.738 mg/100 g DM 5-Methyltryptamine, present at a concentration of 3.99 mg/100 g DM in the mycelium of *F. betulina*, is virtually not noted in the fruiting bodies of *L. edodes* and *Leccinum scabrum* [33]. l-Tryptophan and 5-hydroxytryptophan have a sleep-depriving effect and support the treatment of depression. They are also precursors of serotonin and melatonin—endogenous substances responsible for regulating the circadian cycle of our body. Serotonin also has proven antioxidant and anti-cancer properties.

**Fig. 1:**
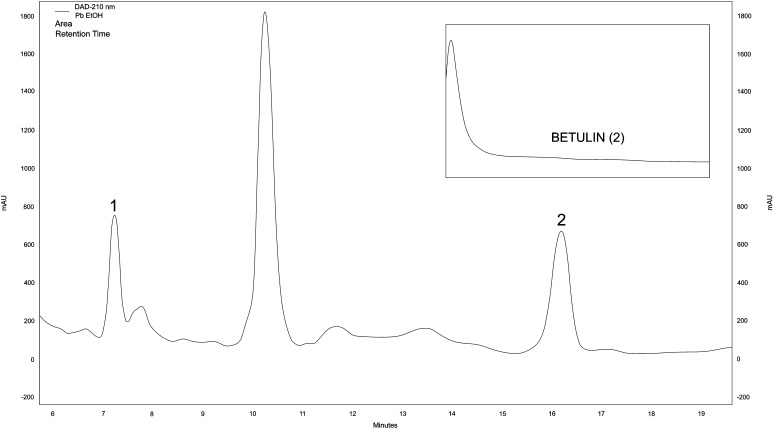
HPLC chromatographic separation of triterpenoids from mycelial culture of 1—betulinic acid, 2—betulin. UV spectrum of betulin

Chromatographic analysis showed the presence of two peaks characteristic of betulin and betulinic acid. HPLC chromatogram of triterpenes separation in mycelial cultures shows Fig. 1. In fruiting bodies, the content of betulin and betulinic acid was 0.0006 and 0.00031 mg/100 g DM, respectively, while in mycelial cultures it was 0.00209 and 0.00085 mg/100 g DM (Table 1). In addition, betulin was isolated and spectrophotometrically identified in biomass. The existence of betulin was confirmed by the presence of the following signals: ^1^H NMR (300 MHz, CHLOROFORM-d) d ppm 4.68 (1 H, d, J = 2.34 Hz), 4.58 (1 H, dd, J = 2.34, 1.17 Hz), 3.79 (1 H, dd, J = 10.55, 1.76 Hz), 3.33 (1 H, d, J = 10.55 Hz), 3.18 (1 H, dd, J = 10.84, 4.98 Hz), 2.38 (1 H, td, J = 10.70, 5.57 Hz), 1.68 (s), 1.02 (s), 0.98 (s), 0.97 (s), 0.82 (s), 0.76 (s).

For example, in fruiting bodies of another arboreal species of mushroom *Inonotus obliquus*, the content of betulinic acid and betulin was determined at 103.0 mg/g and 1490.0 mg/g respectively [34].

Betulin exhibits a broad spectrum of biological activity with respect to many diseases, including anticancer activity. It interacts with Sterol Regulatory Element-Binding Proteins (SREBP), which participates in the activation of the expression of genes involved in the synthesis of cholesterol, fatty acids and triglycerides [35]. Compounds from the group of terpenoids with anti-inflammatory, antiproliferative and anticancer potential have been isolated from numerous species of mushrooms living on wood. One of these is *Poria cocos*. An anti-inflammatory activity of extracts from this mushroom species has been demonstrated in an in vitro model using RAW 264.7 macrophages. A decrease in the generation of pro-inflammatory mediators by inactivating the NF-κB signaling pathway was observed [36].

In turn, the anticancer activity of fungal terpenoids was tested on the U937 line. The concentration and time-dependent antiproliferative effect and pro-apoptotic effects of the examined extracts is related to the release of cytochrome C to the cytosol, activation of caspases − 3, − 8 and − 9, degradation of PARP and mitochondrial membrane potential loss. The obtained results indicate the potential of *P. cocos* in the treatment of leukemia [37]. A series of triterpenes with a similar inhibitory effect on the NF-κB signaling pathway has been isolated from another species, *Inonotus obliquus* [38]. In turn, the extracts from *Antrodia camphorata* inhibit the proliferation of cancer cells, and also show a reduction of NO, TNF-α and IL-12 [39].

**Fig. 2:**
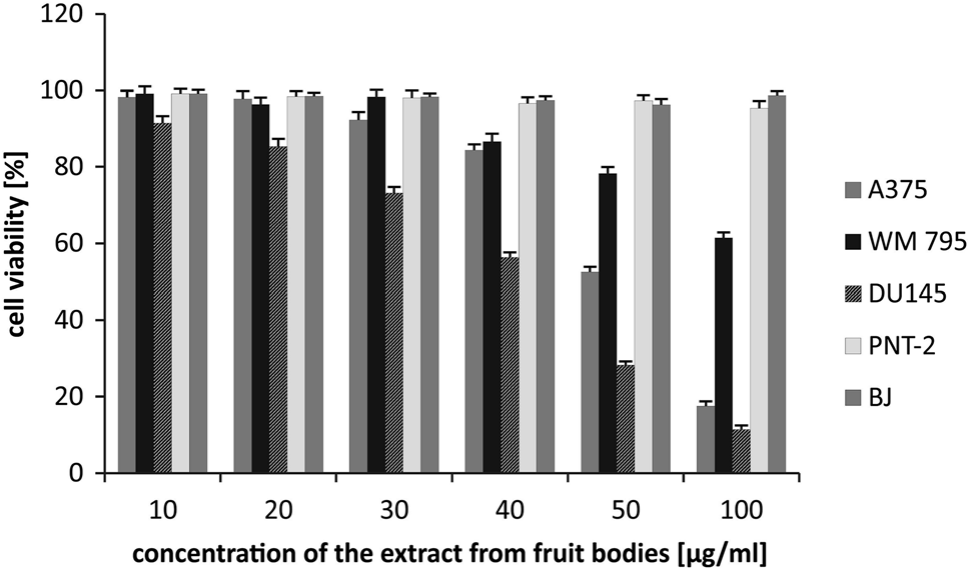
Cytotoxic activity of extracts from fruiting bodies of *Fomitopsis betulina*

**Fig. 3:**
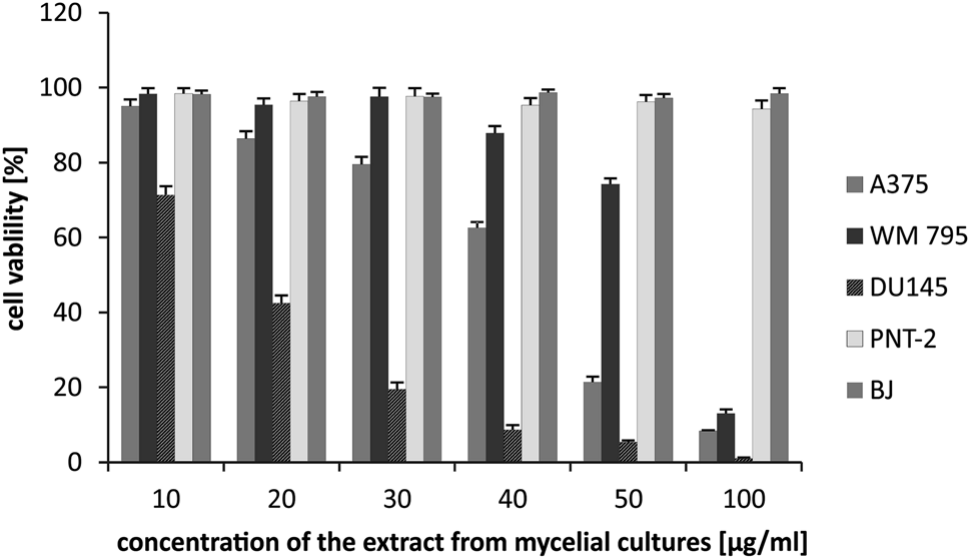
Cytotoxic activity of extracts from mycelial culture of *Fomitopsis betulina*

All examined extracts revealed varied cytotoxic effects against the cell lines used in the experiment. The mycelium extract exhibited significant cytotoxic activity against DU145 prostate cancer cells (27.28 ± 0.98 and 5.37 ± 0.31% of viable cells at 20 and 50 µg/mL, respectively) and a moderate effect was observed against A375 melanoma cell lines (21.39 ± 1.4% of viable cells at 50 µg/mL), while the other melanoma cell line, WM795, seemed to be less vulnerable, with 74.21 ± 1.29% of viable cells at 50 µg/mL. Most importantly, the extract had no cytotoxic effect on normal human skin fibroblasts and prostate epithelial cells, which indicates its selectivity. The results of the cytotoxic effect of fruiting body extracts indicated a moderate influence on the viability of A375 melanoma and DU145 prostate cancer cells (52.12 ± 1.45 and 69.32 ± 1.69% of viable cells at 50 µg/mL, respectively), while no impact was observed on WM795 melanoma cells (96.22 ± 1.63% of viable cells at 50 µg/mL). Normal human skin fibroblasts were unaffected by the extract, but a strong cytotoxic effect was observed on normal prostate epithelial cells (5.21 ± 0.43% of viable cells at 50 µg/mL) (Figs. 2, 3). The cause of differences in the cytotoxicity of extracts obtained from fruiting bodies and mycelial cultures can be related to the different chemical composition of the material in vitro and in vivo. Extracts from fungal cultures possess a higher content of biological active sterols or triterpenoids.

Extracts obtained from fruiting bodies show higher activity, with respect to normal prostate cells, in relation to cultures, while cultures have a stronger influence on melanomas, especially A375, and to a lesser extent WM795. To the best of our knowledge, our study is the first to demonstrate the cytotoxic potential of *mycelial cultures of F. betulina* on prostate cancer and melanoma cells, while we have been able to find only one similar experiment on the cytotoxic activity of the mycelium (i.e. Cyranka et al.) against human colon cancer LS180 and normal CCD 841 CoTr cells. Ether and ethanolic extracts of the tested mushroom revealed selective cytotoxic and antiproliferative activity against cancer cells, with almost no impact on normal colon cells [8].

Some authors have described the impact of *F. betulina* fruiting bodies on a number of murine and human cancer cell lines. Kaczor et al. demonstrated a significant cytotoxic activity of the ether extract on FTC238 human thyroid carcinoma, SK-N-AS neuroblastoma, T47D breast carcinoma, Hep-2 larynx carcinoma and HeLa cervix carcinoma, in a time- and dose-dependent manner, with the most profound effect being for FTC238 cells. Moreover, the tested extract induced cell cycle arrestment in S-phase for thyroid carcinoma cells [40].

In a study by Tomasi et al., the moderate cytotoxic effect of methanolic extract of *F. betulina* fruiting bodies was observed against 3 LL murine Lewis lung carcinoma and L1210 lymphocytic leukaemia cells, with IC50 values of 88.2 and 77.5 µg/mL, respectively [41].

Ethanolic extract from *F. betulina* fruiting bodies was also tested against human colon epithelial cells—CaCo-2 and HT-29—by Doskocil et al., and the results showed that the extract was cytotoxic against HT-29 (IC50 73 µg/mL), but not CaCo-2 cells [42].

Thus, the results of our study are comparable with those of other authors. Moreover, this is the first study to describe the cytotoxic impact of *F. betulina* fruiting bodies against prostate cancer and melanoma cells.

In the A549 cells incubated with extracts from fruiting bodies and mycelial cultures of *F. betulina* was determined using Western Blot technique, the expression of a COX-2 protein associated with the inflammatory process. As an internal control, glyceraldehyde 3-phosphate dehydrogenase (GAPDH) was used.

**Fig. 4:**
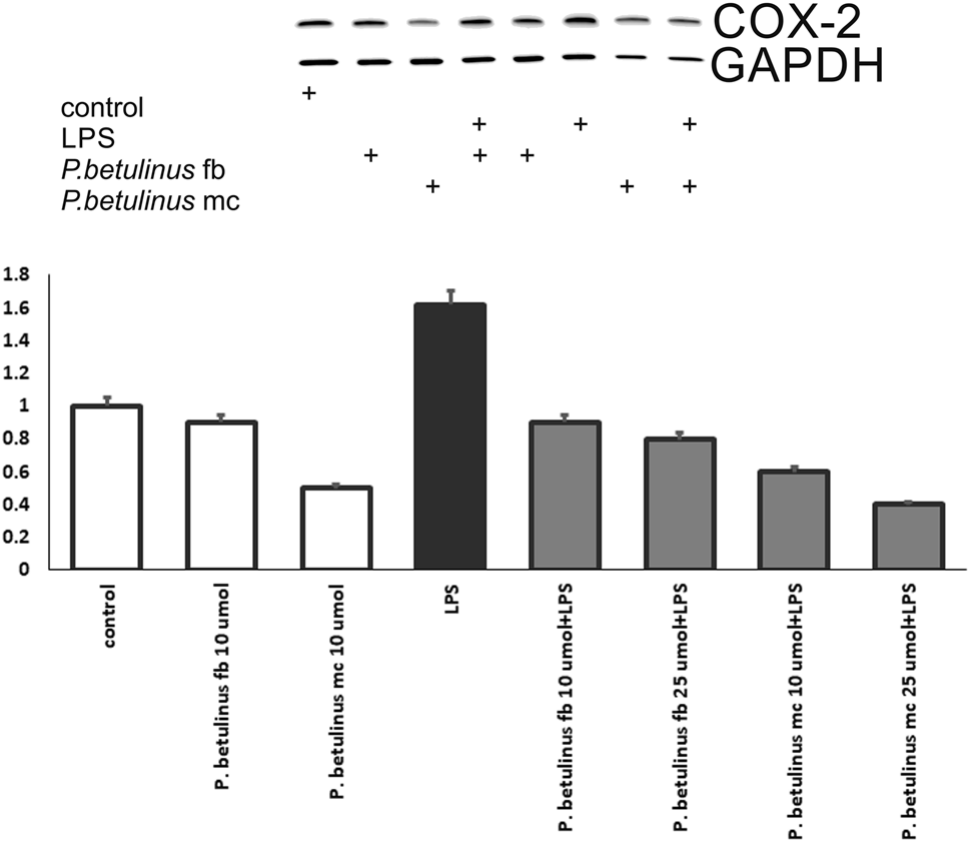
Relative expression of COX-2 in A549 cells supplemented with extracts from fruiting bodies (fb) and mycelial cultures (mc) and activated with LPS

No apoptosis or reduced viability of A549 cells was observed after incubation with fruiting bodies and mycelium extracts in LPS-activated cells. The highest expression of COX-2 was observed in LPS activated A549 cells compared to the control. Cyclooxygenase expression was lower in samples incubated with mycelium extracts of 10 µM and 25 µM activated with LPS compared to extracts from fruiting bodies (Fig. 4).

The primary and secondary metabolites contained in the mushrooms exhibit a beneficial effect on immune system functioning: primarily anti-inflammatory and antioxidant activity. Natural products containing bioactive compounds with the mentioned effect can be used to reduce oxidative damage and inflammation in the human body. Chemical compounds contained in mushrooms also affect the proliferation and differentiation of lymphocyte populations, as well as the migration and adhesion of these cells. They can also inhibit the synthesis of inflammation mediators, i.e. cytokines, interleukins, prostaglandins, or nitric oxide by inhibiting the pro-inflammatory signal pathways associated with NF-κB nuclear receptors [43].

Our study on the anti-inflammatory activity of extracts of both fruiting bodies and mycelium includes anti-inflammatory compounds that can be used in the development of new anti-inflammatory drugs.

Numerous studies indicate that compounds with a lantostanoid structure possess an anti-inflammatory activity. Wangun et al. isolated and fully characterized three compounds with lanostanoid structures which demonstrated cyclooxygenase I inhibition activity, and in addition these compounds proved to be promising anti-inflammatory agents by inhibiting 3-α-hydroxysteroid dehydrogenase. Numerous studies on anti-inflammatory activity have focused on an analysis of extracts obtained from fruiting bodies. Methanol extracts from *Pleurotus pulmonarius* evoke an effect similar to diclofenac, while methanol extract from oyster mushroom causes inflammation of blood vessels [44].

Edible species have been widely studied in terms of anti-inflammatory activity. The study conducted by Muszyńska et al. demonstrated the effect of *I. badia* extracts derived from biomass on simultaneous expression of COX-2, cPGES and GSTM1 proteins and the activity of NF-ĸB and PPARγ transcription factors in RAW 264.7 macrophages. High levels of expression of COX-2 and cPGES2 pro-inflammatory proteins and AHR proteins in LPS activated A549 cells have been found in *C. cibarius* species, and the addition of *C. cibarius* biomass extracts enriched with zinc salts inhibited the expression of the examined proteins [16, 45].

## Conclusion

To the our best knowledge this study for a first time present a comparison of qualitative and quantitative data regarding bioactive secondary metabolites in the fruiting bodies and biomass of *F. betulina*, as well as results of in vitro anti-inflammatory and cytotoxic activity against melanoma and prostate cell lines. Despite records of the use of this species in folk medicine, there has not been any previous characterization of the chemical or pharmacological potential of mycelial cultures. The results of biological activity assays demonstrate that extracts from *F. betulina* show cytotoxic and anti-inflammatory activities at different magnitudes of potency. The mycelial culture extract might be a potential supporting agent in prostate cancer therapy, but further studies are needed.
